# The Impact of Multidisciplinary Research on Progress in Skin Cancer Prevention

**DOI:** 10.3390/cancers17213473

**Published:** 2025-10-29

**Authors:** Alyssa Susanto, Clare Primiero, Simone M. Goldinger, H. Peter Soyer, Monika Janda

**Affiliations:** 1 Frazer Institute, Dermatology Research Centre, The University of Queensland, Brisbane, QLD 4102, Australia; alyssa.susanto@student.uq.edu.au (A.S.); c.bover@uq.edu.au (C.P.); s.goldinger@uq.edu.au (S.M.G.); p.soyer@uq.edu.au (H.P.S.); 2 Faculty of Medicine, University of Zurich, 8032 Zurich, Switzerland; 3 Department of Dermatology, Princess Alexandra Hospital, Brisbane, QLD 4102, Australia; 4 Centre for Health Services Research, The University of Queensland, Brisbane, QLD 4102, Australia

**Keywords:** skin cancer prevention, multidisciplinary research, precision prevention, artificial intelligence in dermatology, data integration and digital health

## Abstract

**Simple Summary:**

Skin cancer is one of the most common cancers worldwide, making its prevention a global priority. Historically, public health and dermatology were focused on primary and secondary prevention, respectively. Merging these efforts resulted in greater impacts. Multidisciplinary research combines the expertise of dermatologists, epidemiologists, behavioural scientists, health economists, geneticists, imaging, and artificial intelligence teams. New tools have helped to improve the accuracy and tailoring of prevention and early detection. However, there are ethical and data protection concerns surrounding their use, alongside unequal access and the need for more reliable long-term evidence. Future research should maintain and expand collaborative relationships, focusing on the fair and equal application of new technologies for use in real-life situations. The involvement of a multidisciplinary team shows potential in reducing the skin cancer burden, as well as contributing to prevention efforts for other health conditions.

**Abstract:**

**Background/objectives**: The global incidence of skin cancer is rising, creating a need to strengthen prevention strategies. In this review, we examine the contributions of public health, dermatology, behavioural science, and emerging technologies such as artificial intelligence and bioinformatics, which have collectively shaped prevention in recent decades. **Methods**: Using a narrative scoping review approach guided by the PRISMA-ScR framework, we synthesised research across these disciplines to highlight their roles in enhancing skin cancer prevention. **Results**: Initial efforts focused on increasing public knowledge through sun protection campaigns and symptom recognition. Dermatologists enhanced early detection through refined techniques and clinical guidelines. Initiatives such as Euromelanoma enabled broader collaboration and population-level screening. As more disciplines joined, advances in risk stratification, digital imaging, artificial intelligence, molecular and genetic diagnostics and bioinformatics became possible. Beyond skin cancer prevention, these tools may have additional applications for systemic health issues. However, a number of challenges remain, particularly regarding data privacy concerns, cost-effectiveness, equitable access, and the validation of artificial intelligence tools in diverse populations. **Conclusions**: The prevention of skin cancer brings together knowledge spanning the fields of public health and dermatology to behavioural research and digital innovation. Working together, these disciplines have improved early detection and awareness. However, fragmented collaboration across regions throughout the world continue to limit their impact. Improved equity alongside stronger, more coordinated partnerships will be essential for the next phase of progress.

## 1. Introduction

Skin cancer prevention and early detection remain major public health challenges. The incidence of both melanoma and keratinocyte cancers (including basal cell carcinoma and squamous cell carcinoma) continue to rise globally with aging populations [1]. This trend has occurred due to a combination of increased disease burden from cumulative ultraviolet (UV) exposure, as well as improved diagnostic tools and reporting practices. Melanoma is still one of the leading causes of cancer-related deaths [2], and keratinocyte cancers impose substantial clinical and economic burdens on healthcare systems [3].

Over recent decades, approaches to skin cancer prevention have expanded beyond traditional education campaigns to include improved behavioural, clinical and technological strategies, collectively representing multidisciplinary collaboration, where dermatologists, public health professionals, behavioural scientists, engineers and policymakers work together to address skin cancer prevention. Unlike earlier siloed efforts, this integrated approach enables broader reach, improved messaging strategies, and scalability to effectively target both individuals and entire populations.

To understand how these strategies have evolved, this narrative scoping review examines the major methods used in skin cancer prevention, including population-based interventions, risk assessment models, imaging technologies and artificial intelligence (AI) tools. We explore both individual- and population-level contributions to skin cancer prevention and highlight how the contributions of many fields across both these domains have transformed prevention from simple awareness campaigns into data-informed, precision-based approaches.

## 2. Methods

This narrative scoping review followed the PRISMA-ScR (Preferred Reporting Items for Systematic Reviews and Meta-Analyses extension for Scoping Reviews) framework [4], integrating literature from a wide range of fields, including public health, dermatology and oncology, epidemiology and biostatistics, behavioural and social sciences, health economics, primary care and implementation research, pathology and genetics, and emerging fields such as AI, imaging, and bioinformatics. We conducted searches primarily through PubMed from inception through September 2025. Further literature was identified through the professional networks and disciplinary expertise of the co-authors. Studies were selected for their conceptual and illustrative relevance rather than formal inclusion or exclusion criteria, and no new data was generated.

## 3. Evolution of Skin Cancer Prevention

Following the scoping review outlined in Section 2, we identified three distinct phases in the evolution of skin cancer prevention: (1) public health initiatives, (2) dermatology-led interventions, and (3) integrated multidisciplinary models. This section synthesises key developments across these phases, highlighting how collaborative approaches have improved outcomes and addressed the limitations of earlier siloed efforts.

### 3.1. Primary Prevention: Public Health Initiatives

Since UV radiation was first recognised as a carcinogen [5], primary prevention strategies have focused on reducing sun exposure and promoting protective behaviours through public education and policy [6]. These efforts, demonstrated by Australian campaigns such as “Slip! Slop! Slap!” [7] and SunSmart [8] in the early 1980s, have demonstrably shifted cultural attitudes away from tanning, particularly in Australia and New Zealand. This also inspired similar campaigns across Europe [9] and the United States [10,11]. Long-term evaluations suggest that public health messaging programs have contributed to improvements in sun protective behaviours and reduced melanoma incidence for those under the age of 40 [12,13]. However, their impact on overall mortality and morbidity varies across populations and timeframes, influenced by factors such as age, unequal access to care, and cumulative lifetime UV exposure [14].

Public health efforts extended into policy, such as complete bans or stricter regulation of sunbed use [15], “No Hat, No Play” requirements in schools [16], and workplace standards mandating the use of sun protection for outdoor workers [6,17]. Health economic studies have since confirmed the cost-effectiveness of these strategies, which resulted in estimated savings of up to $8 for each $1 invested [18,19]. With aging populations and greater access to outdoor recreational activities, the incidence of skin cancer continued to rise, highlighting that early detection is necessary given the long-term effects of sun-exposed activities.

### 3.2. Secondary Prevention: Dermatology-Led Interventions

At the same time as primary prevention initiatives, dermatologists advanced secondary prevention by developing tools such as dermatoscopy [20] and the ABCDE criteria [21,22] to improve melanoma early detection [23]. Dermatoscopy transformed the assessment of pigmented and non-pigmented lesions [20,23]. The ABCDE criteria [21,22] gave patients a practical approach for monitoring their own moles, which strengthened the doctor-patient partnership in early detection. However, concerns regarding their use included variability in diagnostic accuracy [24], over-reliance on clinician judgement [25], and the lack of accessibility in under-resourced clinics [26], all which limited their potential impacts on population-level outcomes.

In 2008, Germany introduced a national skin cancer screening program, showing how dermatology-led strategies could be scaled to the population level. Early data suggested that such a program would reduce melanoma mortality [27]; however, participation rates, cost-effectiveness, and long-term benefits were inconsistent [28]. These limitations highlighted the need to combine clinical expertise and public health infrastructure to achieve sustainable population-level impact.

### 3.3. Integrated Multidisciplinary Models

By the late 1990s, it became clear that siloed approaches to skin cancer prevention were insufficient. Public health campaigns influenced prevention knowledge and attitudes, and dermatology advanced early detection, but neither could fully address the many biopsychosocial determinants of skin cancer risk [29,30]. For example, genetic susceptibility may be influenced by higher UV exposures in certain geographic areas [31]. Behavioural factors such as attitudes towards tanning or sunscreen use could be caused by differences in cultural norms, educational levels, and socioeconomic status [32]. Furthermore, variable access to diagnostic tools and treatment access is likely to cause greater disparities in outcomes [33]. These complex interactions factors highlight how the multifaceted nature of skin cancer raises a need for more integrated strategies that expand beyond isolated interventions.

Since 1999, Euromelanoma [34] has united dermatologists in providing free skin checks across 33 countries, raising awareness, and educating the public. Euromelanoma, and other global initiatives from the United States [35,36] and Australia [37] also generated population data, helping public health to refine messaging strategies for high-risk groups. This movement showed how community influence and clinical expertise together made prevention more effective.

## 4. Multidisciplinary Teams: Roles and Contributions

Although public health and dermatology laid essential foundations for early prevention efforts, broader progress has required input from many other specialties. The combination of diverse skillsets has strengthened prevention strategies, making them more responsive and equitable than traditional, single-discipline models.

### 4.1. Dermatology and Oncology

Joint efforts of dermatologists and oncologists have set the clinical standard for secondary prevention strategies, driven by their shared recognition that survival depends on timely diagnosis. Surgical management remains the cornerstone of early-stage melanoma, with five-year survival rates exceeding 98% [38], while late-stage disease is associated with poorer outcomes [39].

Dermatologists have integrated novel diagnostic imaging tools beyond dermatoscopy [20,23], including confocal microscopy [40] and total body photography (TBP) [41], while advances in dermatopathology led to greater accuracy in histological assessment [42]. Staging guidelines, excision margins, and sentinel lymph node biopsy techniques continue to be refined [43,44], allowing interventions to be both diagnostic and curative [45,46,47]. Mohs micrographic surgery has further enabled complete margin clearance with tissue conservation in selected cases [48].

The most dramatic progress has come from systemic therapy: collaboration between oncologists, basic scientists, immunologists, and the pharmaceutical industry has driven the development of immune checkpoint inhibitors and targeted BRAF/MEK inhibitors [49,50,51], which have transformed outcomes for advanced melanoma, achieving durable remissions and long-term survival in over half of the patients who previously had a dismal prognosis [52]. Despite being initially used only for advanced disease, these agents have rapidly expanded into curative-intent settings, now forming the basis of adjuvant, and more recently, neoadjuvant treatment strategies [53]. These systemic therapies also have implications for secondary prevention due to improved clinical benefits with early detection since timely diagnosis now has potential curative treatment. This may also influence screening strategies in high-risk populations by improving risk-benefit calculations. In addition, supramolecular methods have emerged in cancer therapy, which make use of non-covalent interactions to create drug delivery systems that can overcome biological barriers and deliver highly precise treatment [54,55]. Supramolecular methods may complement existing systemic therapies in the future.

More precise radiation techniques, such as intensity-modulated radiotherapy, stereotactic body radiotherapy, and proton therapy [56], alongside improved imaging-based radiotherapy planning [57], have helped maintain radiotherapy as a useful modality for selected patients. Whilst many of these advanced technologies and procedures across dermatology and oncology may be preferred by patients, the associated costs, concentration in metropolitan areas, and requirements for trained staff and infrastructure are barriers that prevent their widespread uptake [58,59,60]. These equitability and sustainability issues may restrict the ability of these developments to impact broader population-level outcomes.

### 4.2. Biostatistics and Epidemiology

Biostatistics and epidemiology have enabled the identification of individual risk factors by examining patterns and associations over entire populations. Large cohort studies, such as QSkin, which enrolled over 43,000 Queensland participants in its initial cohort, have demonstrated how UV exposure, genetic susceptibility, and behavioural factors interact to influence an individual’s skin cancer risk [61]. Prior to QSkin, prevention strategies were based on small observational studies [62,63,64], limiting their generalisability. The integration of epidemiological expertise has allowed for the development of risk prediction tools designed for more targeted prevention strategies, for example, identifying high-risk individuals for screening [65,66,67].

Although the roles of biostatisticians and epidemiologists may have been overlooked in the past, their contributions are clear. Analytical methods including multivariate modelling and survival analysis have refined clinical guidelines and enabled tracking of long-term incidence trends for subgroups of the population. For instance, multivariate modelling has helped to isolate the effectiveness of sunscreen from confounding variables [68], whilst survival analysis of melanoma registries has shown improvements in outcomes due to early detection [69]. However, limitations of biostatistics include overfitting risk prediction models and reduced accuracy in underrepresented populations [70]. For epidemiology, assumptions made regarding behaviours or access may not be accurate for those who are culturally diverse or socioeconomically disadvantaged [71]. Therefore, ongoing refinement in biostatistics and epidemiology is necessary to ensure equity across all demographic groups and ensure accuracy of population-level insights.

### 4.3. Behavioural Research, Social Sciences and Psychology

Behavioural and social science research has shown that awareness on its own does not always lead to sustained behavioural changes. Cultural norms around tanning, convenience, and perceived low risk all contribute to how much people will protect themselves in certain situations [72,73,74,75]. In response, prevention strategies are now increasingly targeted, for example, appearance-based messaging for adolescents [76,77], cancer-focused and situational alerts for older adults [78], and culturally adapted programs designed to engage Indigenous and migrant communities [79,80]. More recently, digital tools such as web-based programs, mobile applications, and social media, have helped to extend both the reach and personalisation of these approaches [80,81].

Psychological factors add another important layer. Fear of diagnosis [82], burden of treatment, or concerns about body image may lead to anxiety or depression, especially among adolescents [83], indicating a need for increased mental health support. The behavioural and social sciences, in combination with psychology, have helped strategies become more human-centred, adaptable, and culturally responsive.

### 4.4. Health Economics and Health Services Research

As prevention efforts expanded and showed success, health economists and health services researchers questioned whether and how these strategies could be delivered sustainably and fairly. Economic analyses consistently showed strong returns. For instance, the SunSmart program has an estimated return on investment of approximately $8.70 for every $1 spent and expected savings of $63.9 million for the Western Australia economy [19]. Similarly, sunscreen use in high-risk populations showed a 100% probability of being cost-effective compared to early melanoma detection [14]. These findings provided convincing evidence for policy action, such as sunbed bans and workplace safety standards. As advanced cancer treatment becomes more expensive with new targeted immunotherapies [84,85,86], health economic evaluations will likely become even more critical.

However, disparities by geographic location, socioeconomic status, and health system capacity influenced those who benefited most [87]. In response, health services researchers tested new delivery models, including screening in primary care, fly-in fly-out workers, training non-specialists to recognise suspicious lesions, and strengthening follow-up pathways [88,89]. Moreover, the allocation of resources between prevention and treatment raises important ethical considerations. Whilst prevention may yield more favourable cost-effectiveness ratios and population-level benefits [85], treatment on the other hand is medically necessary, time-sensitive and potentially lifesaving [90] for those already affected by skin cancer. Ethical trade-offs occur when either prevention or treatment is prioritised, especially in underserved communities where there is limited access to both [91]. There remains is a strong need for policymakers to balance cost-effectiveness with fairness, equity, and moral obligations, ensuring that all people, regardless of their background, can benefit from prevention and early detection programs.

### 4.5. Primary Care and Implementation Sciences

Primary care, nursing, other allied health professionals and professionals who see a lot of skin such as hairdressers, massage therapists and others working together is essential to prevention program success. General practitioners already provide opportunistic skin checks, risk counselling, and education, and often serve patients in areas where access to secondary or tertiary care is limited [92]. Nurses have expanded their roles to include patient education and follow-up [93], and pharmacists can reinforce sun protection in their daily interactions [94].

Implementation science has developed tools to improve the efficiency of integrating research-tested interventions into practice [95], including decision-support systems [96], teledermatology training [97], and risk-stratified care models [98]. Culturally tailored programs also promote equity, particularly for First Nations peoples, migrants and rural communities [99,100]. Together, these developments have embedded prevention more firmly into routine care.

### 4.6. Pathology and Genetics

Pathologists make important contributions to improving skin cancer outcomes and research into the value of primary and secondary prevention, as histopathology remains the gold standard for diagnosis. However, recent discoveries of immunohistochemical and molecular markers have further clarified how premalignant lesions progress to invasive disease [101,102]. Somatic mutations including *BRAF* [103], *NRAS* [104], and *KIT* [105] provide useful information regarding prognosis and management. Combined with epidemiological data, these findings support personalised prevention that can be uniquely tailored to inherited and acquired risk [106].

The identification of high-penetrance genes linked to hereditary melanoma such as *CDKN2A* [107] and *POT1* [108] have enabled more opportunities for genetic counselling and improved risk stratification [109]. Furthermore, international collaborations such as GenoMEL [110] have linked genotype-phenotype data, providing greater support for precision prevention models [111]. Examples of genomic-informed programs involve individuals with pathogenic variants in *CDKN2A* [112] or *POT1* [113], who are at elevated risk for melanoma. These expert guidelines recommend annual or more frequent full skin examinations as early as age 10 [112] or 20 [113], with adjunctive use of mole mapping or TBP [112] to monitor lesion changes over time. Behavioural counselling focuses on strict photoprotection, regular skin self-examination, and family risk communication [112,113].

Importantly, these genetic findings have limited use in general population screening due to their rarity and complexity in interpreting variants of uncertain significance [114]. In addition, genetic testing is typically only for individuals with a strong family history or suggestion of hereditary melanoma syndromes [115]. From an ethical perspective, genetic counselling is needed to support informed consent, insurance implications, employment, privacy, and testing in family relatives [115]. This is especially relevant in countries like Australia where comprehensive legal protections against genetic discrimination, including life insurance, is lacking [116]. Further ethical issues regarding autonomy, psychological impacts, and timing of disclosure are relevant for testing in minors [117]. As technologies become more accessible, genetic testing should remain clinically justifiable and ethically sound with adequate psychosocial support provided.

### 4.7. Computational Science, Imaging, and Artificial Intelligence

Imaging has become one of the most important new tools for skin cancer prevention and early detection. Dermatoscopy [20,23], confocal microscopy [40], and 3D-TBP [118], have revolutionised how lesion monitoring, risk assessment, preventive counselling and early detection are conducted.

These advances have been strengthened by integrating experts in computational science, imaging analysis, and AI methods [119]. Recent studies have shown that AI algorithms can match or exceed expert-level performance in skin cancer detection, particularly in reader studies using curated dermatoscopic datasets such as the International Skin Imaging Collaboration (ISIC) Archive [120]. However, results from clinical workflow studies have been more variable, and many models are limited due to the underrepresentation of skin of colour [121,122]. For example, AI models trained on predominantly light skin types have a higher rate of misclassified lesions on darker skin tones [123], emphasising these inequities.

Validation is also a challenge, given that many AI tools have not been tested in real-world clinical settings outside of controlled environments [124]. Regulatory standards are still evolving, and questions remain about how to ensure seamless clinical integration and how to track outcomes over time [124]. To help readers critically assess the readiness of AI tools for clinical use, we have included a checklist adapted from the TRIPOD+AI [125] and DECIDE-AI [126] reporting standards, which reflects the current best practices for reporting, validation, and implementation of AI in healthcare (Table 1).

Clinically, AI tools can show the degree of sun damage on the skin, prioritise lesions for review, highlight lesion changes in sequential imaging, guide decisions on follow-up intervals, and extend equitable care to communities with limited specialist access [127,128,129,130,131]. At the same time, these innovations raise valid concerns. Protecting patient privacy, maintaining accountability, and clinical judgement are essential. Thus, the use of imaging and AI require care and transparency, guided by ethical and professional standards [132].

### 4.8. Bioinformatics and Data Integration

The rapid growth of registries, imaging archives, and genomic studies has highlighted the challenges of data integration. Bioinformatics, through linkage of these diverse resources, has made predictive modelling, risk stratification, and long-term surveillance possible.

**Figure 1:**
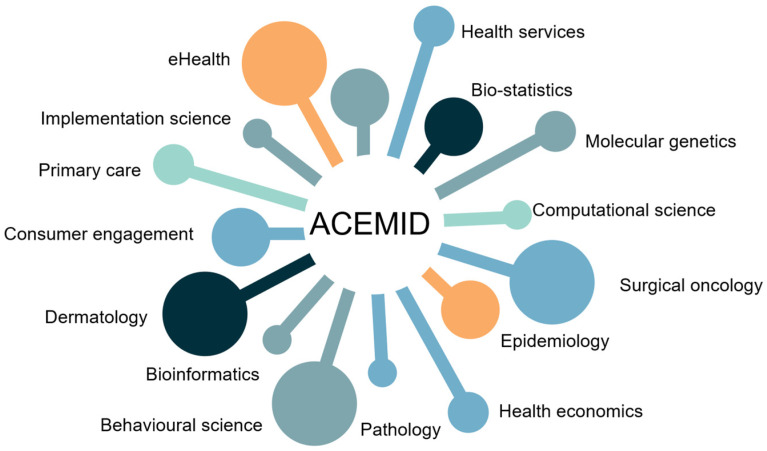
ACEMID team capability framework, illustrating the central role of ACEMID and its multidisciplinary teams.

The Australian Centre of Excellence in Melanoma Imaging and Diagnosis (ACEMID) provides a leading example (Figure 1) [133,134]. By combining 3D-TBP, clinical data, and genomic testing into a national platform, ACEMID has concurrently advanced AI algorithms, while seamlessly integrating them into multiple hospital and health care settings, learning important health services lessons along the way [135,136]. In addition to ACEMID, several global infrastructures have advanced research and innovation in skin cancer prevention and diagnosis. The ISIC Archive [103] provides open-access dermatoscopic image datasets supporting AI development for melanoma detection, while national registries such as the United States Surveillance, Epidemiology, and End Results (SEER) Program [137] supply epidemiological data for population-level surveillance. Together, these efforts reinforce the international framework for skin cancer control (Table 2).

Similarly, international collaborations are being remodeled by bioinformatics. The use of federated learning, cloud-based platforms, and cross-border networks has allowed researchers to share findings with collaborators across the world and align their methods to accelerate scientific discovery [138]. This in turn has made bioinformatics the infrastructure that connects and sustains multidisciplinary research.

## 5. Technology and Innovation: Shaping the Future

The translational trajectory of skin cancer prevention has moved scientific discoveries from bench to bedside whilst maintaining scalability. This can be seen through the rise of specialist training programs with joint fellowships [139], preparing future health professionals who will have to work across disciplines and building the collaborative capacity required for this next phase.

Unfortunately, the path from bench to bedside is rarely straightforward. New technologies and models often create significant barriers. High set-up expenses are often associated with AI tools [140], and regulations surrounding data privacy and validation can cause further delays [141]. Some clinicians may be reluctant to accept these new tools if they use unfamiliar systems or alter established workflows [142]. Unless these barriers are addressed early, even the most promising technologies risk underuse. We have provided a forward-looking toolkit for stakeholders, summarising methods that could be used to model future prevention scenarios, arranged from lowest to highest fidelity (Table 3). At the lower end, expert consensus and epidemiological modelling offer broad projections of risk and intervention impacts. Mid-level approaches of behavioural interventions and implementation trials test their feasibility in real-world settings. Finally, high-fidelity computational tools, including microsimulation modelling, digital twins and AI-driven prediction platforms, create dynamic, increasingly personalised forecasts of individual skin health.

The concept of “cutaneous digital twins” [143], computational models representing an individual’s skin in real time, is an exciting progression in skin cancer research. Through a combination of imaging, demographic, behavioural, and molecular data, these models can simulate how the skin reacts under different exposures and interventions. In practice, measurable outputs include naevus evolution tracking and UV vulnerability mapping, with practical data sources such as serial dermatoscopic imaging from the ISIC Archive [120] and ACEMID [134]. An early version of this has already shown promise with encouraging preventive behaviours in high school students [144].

Dermatological imaging tools can even be repurposed for uses beyond the skin. The ACEMID study has demonstrated how 3D-TBP can be leveraged to derive anthropometric measures such as body mass index, waist circumference, and android/gynoid fat distribution [145,146]. These metrics, when linked to cardiometabolic risk scores, offer measurable outputs for systemic health prevention, with ACEMID’s longitudinal dataset serving as a practical data source [134]. Given the association between obesity measures and cardiometabolic risk, existing dermatology tools are likely to become important in systemic health prevention. Despite this, although early studies show promise, accuracy can be affected by factors such as body posture, lighting conditions, and image quality. These tools should therefore be used as adjuncts, rather than replacements for comprehensive clinical assessment, including medical history, laboratory testing, and broader cardiometabolic assessment [146]. Moreover, there is potential for misuse, especially if AI-derived metrics are interpreted in the absence of appropriate clinical context, such as in consumer-based applications or wellness platforms.

However, these advances are not always equitable. An example is the widespread underrepresentation of individuals with skin of colour in dermatology research [147], particularly in training datasets for machine learning algorithms [148]. Melanoma in skin of colour typically presents differently (e.g., in acral sites), and often at more advanced stages [149]. These disparities are worsened by limited access to dermatologists, underrepresentation in clinical trials, and the absence of inclusive design in technology development. If no deliberate correction is made, this risks the perpetuation of inequalities in skin cancer research [147]. Algorithm auditing, inclusive data collection, and culturally sensitive methodologies are to ensure that technological innovations benefit all groups equally.

Different fields contribute to unique measurable outputs. For epidemiology, this includes age-standardised incidence and mortality rates from practical data sources such as SEER [137] and the Australian Cancer Database [150]. Outputs in behavioural science include sun protection adherence scores that can be obtained from population surveys and wearable UV sensors [151]. Health economics provides measurable cost-effective ratios or return-on-investment metrics, which can be calculated through Medicare claims data or program-level expenditure reports [19,152]. Implementation science offers uptake and fidelity metrics sourced from clinic-level audits and electronic health records [91,153]. When integrated with digital twin technologies, these disciplines enable personalised prevention strategies. We have summarised these measurable outputs, practical data sources, and associated equity and diagnosis metrics (Table 4) to guide service leads and policy teams in evaluating and adapting prevention strategies for diverse populations.

The current literature shows that technology is not necessarily replacing multidisciplinary research but instead extending its reach. The future of prevention requires collaboration between teams to effectively integrate and adapt new technologies to meet the needs of populations and individuals. As prevention strategies become increasingly complex and technology-driven, effective collaboration requires a diverse skillset from each discipline. Key skills are required within multidisciplinary teams to ensure that equitable and sustainable health outcomes are achieved (Table 5).

## 6. Gaps, Controversies, and Challenges

Multidisciplinary research has achieved renewed focus on skin cancer prevention; however, important gaps remain. The translation of precision prevention into practice depends on stronger, long-term evidence. Randomised trials and health economic assessments are necessary to confirm the value of technologies like AI diagnostics, genomic risk mapping, and risk-adapted screening [135,154]. At present, small sample sizes, limited follow-up, and variable study methods restrict both interpretability and reproducibility across populations [128].

Furthermore, increased diagnostic sensitivity also raises issues of overdiagnosis and overtreatment, which have uncertain benefit for long-term patient outcomes. To address these concerns, we have also incorporated equity and overdiagnosis metrics into our summary framework (Table 4), and advocate for future studies to prioritise rigorous methodologies, longer follow-up periods, and more inclusive recruitment strategies to optimise the effectiveness and equity of precision prevention strategies.

Even well-established prevention components, such as sunscreens, remain subject to debate. Although decades of evidence support their role in skin cancer risk reduction [155,156,157], uncertainties around the effectiveness, long-term safety, and adequacy of current regulatory testing have recently been raised. These issues prompted the creation of international workshops that brought together experts from dermatology, epidemiology, policy, pharmaceutical formulation, and public health [158]. Their conclusions emphasise the importance of ongoing assessment, and the search for new and more precise laboratory methods, to ensure they can be used by the population with certainty.

The unequal distribution of new technologies continues to limit timely access to skin cancer prevention. Metropolitan centres often benefit from a higher concentration of dermatologists and imaging specialists, whereas rural and disadvantaged areas struggle with limited services [159]. These inequities apparent in under-resourced settings are reinforced by inadequate health policies and the absence of stable funding for teledermatology services [160,161], leading these systemic issues to potentially limit progress and delay early diagnosis.

Another barrier is the lack of reliable biomarkers to guide individualised risk assessment [162]. In response, input from social sciences, primary care, and policy input are needed to design strategies that ensure equitable and cost-effective access. This may include expanding training programs for rural clinicians, integrating teledermatology into national screening programs, and focusing on funding models that prioritise inclusive technology development.

The use of AI in healthcare requires the collaborative efforts of ethicists, legal experts, and patient advocates, to instill public trust and responsible implementation [163]. If not handled appropriately, data breaches have the potential to expose sensitive patient information [164]. Algorithmic bias shows potential to widen existing health disparities [165]. As legal frameworks are still in development, it leaves many clinician and patient questions surrounding accountability, consent, and liability unanswered [166]. Therefore, there is a need for transparent, consistent, and inclusive governance.

Climate change adds a further layer of complexity to skin cancer prevention. For example, Australia has seen an average temperature rise of 1.44 °C since 1910 [167], which has contributed to people’s behaviour when outdoors, leading to an increased intensity of sun exposure [168]. Combined with well-established ozone depletion and atmospheric changes [169], elevated cumulative UV exposure will likely impact future skin cancer trends. In addition, urban heat islands may amplify the intensity of local UV radiation, most pronounced for dense populations with limited shade access [170]. Public health communication should undergo updates to better reflect the dynamic nature of UV risk, and access to real-time data obtained through the free SunSmart Global UV app [171] could help individuals improve tailoring of their sun protection. Similarly, outdoor workers could benefit from shade, protective clothing, flexible scheduling, and rest breaks during peak UV periods [172]. Considering climate change in prevention strategies is necessary for their effectiveness amidst the ongoing challenges of global warming.

Debates also continue around population versus targeted screening. Germany’s national program has shown mixed mortality outcomes [28] in contrast to opportunistic screening in countries such as Australia, the United States, and the United Kingdom, offering flexibility at the expense of inequity, inconsistency, and lack of quality assurance. Future prospective studies are needed to define the optimal frequency and modality of screening for high-risk groups.

From the patient’s perspective, skin cancer prevention has undergone substantial changes. Historically, dermatologist visits used to be for individuals who noticed visible or progressive lesions on their skin, and delayed diagnoses were common. Today, current population-level campaigns [34,35,36], digital risk assessment tools [30], and TBP [41] have all facilitated earlier patient engagement, with skin checks viewed as a more preventive measure. Genetic testing [109] and AI tools [124] raise questions regarding resource allocation, specifically how individuals should be identified as high-risk, enrolled in registries, or included in behavioural studies. Importantly, psychosocial therapies [173] have helped address anxiety surrounding surveillance, provide reassurance after benign results, and even manage distress related to overdiagnosis. By incorporating psychological therapies into patient management, it has the potential to improve adherence to sun protective behaviours, reduce potential barriers to avoidance, and overall contribute to lower skin cancer incidence on both an individual- and population-level in the future.

## 7. Conclusions

This narrative scoping review shows that a much wider range of disciplines are now involved in skin cancer prevention. While partnerships between public health and dermatology have expanded the scope of prevention, the impact of these collaborations on accuracy, accessibility, and effectiveness remains promising but still inconclusive.

The development of new technologies also raises challenges in how they should be effectively integrated into routine patient care. Algorithmic bias, regulatory uncertainty, and clinical validation pose issues towards the use of AI lesion detection algorithms. Despite the promise of imaging, substantial infrastructure and workforce changes are necessary before equitable access can be achieved. Issues of cost, interpretation, and ethical considerations for genetic testing are especially relevant in the context of population-level screening.

Equity-focused strategies are required to actively address these health disparities, including inclusive data collection protocols, culturally sensitive public health messaging, and workforce development focused on underserved areas. Skin cancer prevention is therefore not only a global priority, but it also can be a model for progress in modern medicine, demonstrating how diverse disciplines can work together to transform innovation into inclusive real-world impacts.

## Figures and Tables

**Table 1:** AI readiness checklist for skin cancer detection tools, adapted from the TRIPOD+AI and DECIDE-AI reporting standards.

Criteria	Considerations
Study design	Was the AI tested in a reader study or clinical workflow?
Dataset composition	Was there adequate representation of skin tones, lesion types, and demographics?
External validation	Was the model validated on independent datasets or in different clinical settings?
Human-AI interaction	Was clinician input part of the workflow, and if so, how did it affect outcomes?
Reporting transparency	Have model architecture, training data, and performance metrics been clearly reported?
Equity and bias	Were any steps taken to address underrepresentation of skin of colour?
Implementation readiness	Did the tool meet clinical usability, safety and regulatory standards?

**Table 2:** Global infrastructures supporting skin cancer prevention. This table highlights some key parallel infrastructures that operate alongside centre-specific initiatives such as ACEMID.

Infrastructure	Example	Description
International datasets	ISIC Archive [120]	Large annotated dermatoscopic image dataset used to train and validate AI algorithms for melanoma detection
National registries	SEER Program [137]	Population-level data supporting cancer surveillance, screening, and epidemiological research
Collaborative research networks	Euromelanoma [34]	Multinational initiative promoting awareness, education, clinical research, and policy development

**Table 3:** Examples of methods to model future prevention scenarios. From left to right, approaches are ordered from lowest fidelity to highest fidelity.

Method	Expert Consensus	Epidemiological Modelling	Behavioural Simulation	Health Economic Simulation Models	Implementation Studies	Digital Twins	Integrated AI-Driven Prediction Platforms
Description	Narrative synthesis or expert panels for future needs	Statistical models projecting incidence, mortality, and outcomes	Experimental or digital studies testing behavioural responses	Cost-effectiveness analyses predicting sustainability	Real-world testing of workflows and delivery of care	Virtual models of individuals or populations for prevention and surveillance	Microsimulation modelling, multimodal data integration to forecast outcomes
Example	Guidelines on risk stratification, national screening	QSkin projections of melanoma trends with changing UV exposure	Appearance-based messaging, smartphone reminders	Cost evaluations of banning sunbeds and opportunistic screening	Teledermatology for rural settings, opportunistic screening by nurses	Longitudinal mole monitoring, optimised screening intervals	ACEMID predictive models linking melanoma risk with cardiometabolic outcomes

**Table 4:** Summary of impacts across different skin cancer prevention domains.

Domain	Measurable Output(s)	Practical Data Source(s)	Equity Metric	Overdiagnosis Metric
Epidemiology	Age-standardised incidence and mortality rates	National cancer registries (e.g., SEER, Australian Cancer Database)	Incidence by skin type, age, geographic location	Stage distribution at diagnosis
Behavioural science	Sun protection adherence scores	Population surveys, wearable UV sensors	Behaviours stratified by socioeconomic status, education, ethnicity	N/A
Health economics	Cost-effectiveness ratios, return-on-investment metrics	Medicare claims data, program-level expenditure reports	Cost–benefit across income quintiles	Cost per additional diagnosis
Implementation science	Uptake and fidelity of interventions	Clinic-level audits, electronic health records	Intervention reach by rural status and language access	Fidelity versus unnecessary follow-up rates
Digital twins/AI	Naevus evolution tracking, UV vulnerability mapping	ISIC Archive, 3D-TBP imaging datasets (e.g., ACEMID)	Model performance of AI across different skin types	False positive rate in low-risk populations

N/A: not applicable.

**Table 5:** Examples of key skills required by multidisciplinary teams.

Clinicians	Researchers	AI Developers
Knowledge of the type and use of imaging analysis and AI methods	Statistical methods to assess value of AI model, clinical applications of AI	Which clinical tasks are repetitive and challenging for clinicians and would benefit from AI support?
Real-world applications of AI	Processing large datasets, including data management	Bias associated with datasets due to selection of clinically notable lesions
Which clinical tasks are highly suitable for AI algorithm support?	Key issues with AI validation and how to assess generalisability of training and test datasets	Understanding of diagnostic assessment for images, so that they can be modelled
Know the strengths and weaknesses of various AI and machine learning models (e.g., supervised versus unsupervised methods)	How to interpret training/testing metrics, and explain the AI decision making process	Understand whether model is overfitting or underfitting given its clinical purpose, and select appropriate threshold

## Data Availability

No new data were generated or analysed in this narrative review. The findings are based on the synthesis of previously published literature, which can be accessed by readers through the citations provided within the manuscript.

## Abbreviations

The following abbreviations are used in this manuscript:

3D-TBP	Three-Dimensional Total Body Photography
ACEMID	Australian Centre of Excellence in Melanoma Imaging and Diagnosis
AI	Artificial intelligence
ISIC	International Skin Imaging Collaboration
SEER	Surveillance, Epidemiology, and End Results
TBP	Total Body Photography
UV	Ultraviolet
